# Crimean-Congo hemorrhagic fever with hepatic impairment and vaginal hemorrhage: a case report

**DOI:** 10.1186/s13256-018-1665-4

**Published:** 2018-05-04

**Authors:** Ermira Muco, Najada Como, Siva Bino, Arjan Harxhi, Pellumb Pipero, Majlinda Kota, Jonida Mehmeti, Arta Kushi, Dhimiter Kraja

**Affiliations:** 1 Service of Infectious Diseases, University Hospital, Tirana, Albania; 2 Institute of Public Health, Tirana, Albania; 3 Infectious Disease Service, Hospital of Saranda, Sarende, Albania; 4 Departament of Infectious Diseases, University Hospital, “Mother Tereza” Tirana, Dibra Street, No 370, Tirana, Albania

**Keywords:** Crimean-Congo hemorrhagic fever, Hypertransaminasemia; vaginal hemorrhage, Hepatic encephalopathy, Ribavirin

## Abstract

**Background:**

Crimean-Congo hemorrhagic fever is a tick-borne disease described in more than 30 countries in Europe, Asia, and Africa. Albania is located in the southwestern part of the Balkan Peninsula. In 1986, the first case of Crimean-Congo hemorrhagic fever was registered, and cases of patients with hemorrhagic fever are rising, and most of them present in a serious condition, when the mortality rate is very high. In districts like Mirdite, Lezhe, Gjirokaster, Skrapar, Erseke, and Kukes, there is delineated human-to-human transmission.

**Case presentation:**

We report the case of a 32 year-old Albanian woman from a rural area of Albania. She was hospitalized at the Infectious Diseases Service, for a severe influenza-like illness of 4 days duration. Our patient had been bitten by a tick while working in her garden. She presented with nausea, vomiting, headache and muscle pain. A physical examination found a high fever of 40 °C, an enlarged liver, petechia, and vaginal bleeding; flapping tremor and fetor hepaticus were found as a sign for hepatic encephalopathy; and confusion and disorientation were observed in her neurological examination. Her platelet and white blood cell counts were very low, while her aspartate aminotransferase and alanine aminotransferase levels were very high. She was transferred to the intensive care unit because of her worsening condition. Serological and C-reactive protein test results for Crimean-Congo hemorrhagic fever were positive. She was treated with oral ribavirin and discharged with normal parameters.

**Conclusions:**

People in high-risk professions in the endemic areas should be informed and trained on the risk of Crimean-Congo hemorrhagic fever as a matter of urgency. Vaginal bleeding is not always a gynecological problem. In Albania, these places are the mountainous areas, so people who have traveled to these areas and who have symptoms after a tick bite are advised to contact their doctors.

## Background

Crimean-Congo hemorrhagic fever (CCHF) is a tick-borne disease caused by the arbovirus Crimean-Congo hemorrhagic fever virus (CCHFV). It occurs in about 30 countries worldwide and it has the most extensive geographic range among the significant tick-borne viruses [1]. The virus is a member of the Nairovirus genus, Bunyaviridae family. CCHF virus is transmitted to humans by tick vectors, Hyalomma marginatum, or by direct contact with the blood of infected humans or domestic animals, causing severe disease in humans, with reported mortality rates of 15–70% [2]. Sporadic outbreaks of CCHF have previously been recognized in Asia, Africa, the Middle East and Europe but, in the 21st century, outbreaks have become more frequent in former Yugoslavia, Turkey, and Iran [3]. Albania is part of Balkan Peninsula, located in the southwestern part. Since 1986, when the first case was registered, cases of patients with hemorrhagic fever have been rising, and most of them present in a serious condition, when the mortality rate is very high. In districts like Mirdite, Lezhe, Gjirokaster, Skrapar, Erseke, and Kukes, there is delineated human-to-human transmission.

We describe the epidemiological, clinical, and laboratory findings, and the role of oral ribavirin therapy for a patient who was diagnosed Crimean-Congo hemorrhagic fever (CCHF).

## Case presentation

On T = 0, a 32-year-old Albanian woman from a rural area in Kukes, Albania, was hospitalized at the University Clinic of Infectious Diseases in Tirana, Albania. Our patient started complaining 3 days after being bitten by a tick while working in her garden (our patient was working with animals). The time period between the onset of symptoms and referral to the hospital was 4 days. This was a case of a patient with no history of traveling to another CCHF-endemic area. The signs and symptoms were nausea, vomiting, headache, myalgia, and fever of 40 °C. The physical findings included hepatomegaly, petechia, metrorrhagia, (without gynecological history). The cardiac and respiratory examination showed hypotension, relative bradycardia, and tachypnea. Flapping tremor and fetor hepaticus were found, indicating hepatic encephalopathy. Central nervous system dysfunction presented confusion and disorientation. No renal failure was observed in the laboratory tests. There was hematuria (3.3 g) in her urine sample. Initial laboratory values were remarkable for thrombocytopenia and showed elevated liver enzymes. Her erythrocyte sedimentation rate was 32 mm/h. Her platelet count was 27,000/mm^3^, white blood cell count was 1,300/mm3, and hemoglobin 11.5 gr/dL. Her aspartate aminotransferase level was 3650 IU/L, alanine aminotransferase level was 1767 IU/L, and bilirubin level was 2.7 mg/dL. The second day, she was transferred to the intensive care unit because of her worsening condition. CCHFV was detected from a blood sample drawn on the second day with a positive enzyme-linked immunosorbent assay (ELISA) test for specific CCHF immunoglobulin M (IgM) antibodies and isolation of the CCHF genome by nested reverse transcriptase C-reactive protein (CRP).

The test results for antinuclear antibodies, anti-smooth muscle antibodies, and anti-liver-kidney antibodies were negative. HbsAg results were positive while, anti-Hepatitis A Virus antibodies (anti-HAV) IgM, Hepatitis B core (HBc) IgM, HBc IgG, Hepatitis C Virus (HCV) and anti-Hepatitis E Virus (HEV) were negative. A direct Coombs test was negative. Toxic hepatitis was excluded. Serological testing was performed for detection of Leptospira, Salmonella, Rickettsia, Brucella, and Toxoplasma species, agents of Lyme disease, herpes, and cytomegalovirus and came out negative. Oral ribavirin was administered within a mean of 10 days after the onset of symptoms at the dosage recommended by World Health Organisation (WHO) (30 mg/kg as an initial loading dose, then 15 mg/kg every 6 h for 4 days, and then 7.5 mg/kg every 8 h for 6 days). The intravenous form of ribavirin was not available in Albania. The patient was hospitalized for 22 days.

Details of the laboratory progress of our case during her hospital stay are in Table 1.

**Table 1:** Laboratory data of biochemical and clinical tests

Laboratory data	Reference range	18.06	19.06	20.06	22.06	24.06	28.06	03.07	07.07
AST	0–40 U/L	6250	8260	7850	1023	343	97	42	39
ALT	0–40 U/L	3150	4460	3380	1411	821	422	145	84
Total bilirubin	< 1.2 mg/dL	2.9	6.7	8.1	12.2	9.4	8.3	5.2	3.8
PTT	60–70s	27.3	23.5	32.3	56.7	74.4	92.9	101	107
Glycemia	70–100 mg/dL	321		273	246	211	160	134	91
Platelet count	150–400 × 103	39	51	70	76	127	138	200	240
WBC	4000–10,000/mm3	1300	2100	2200	4900	4800	6700	8100	9200

*AST* aspartate aminotransferase; *ALT* alanine aminotransferase, *PTT* protrombine index, *WBC* white blood cell count

## Discussion

CCHF is a severe tick-borne illness caused by the CCHFV. The presence of various tick-borne pathogens such as Crimean-Congo hemorrhagic fever virus in various tick species has been documented [4]. Exposure to ticks or direct contact with virus-infected animal are considered major risk factors, therefore, CCHF occurs most frequently among agricultural workers following the bite of an infected tick, and, to a lesser extent, among slaughterhouse workers exposed to the blood and tissues of infected livestock and medical personnel through contact with the body fluids of infected patients [5]. Changes in climatic conditions have been suggested to be one of the factors that has facilitated the reproduction of the tick population, and consequently, the increased incidence of tick-borne infectious diseases. CCHF is a seasonal and clinical overlap among the four diseases (Hantanviruses, leptospirosis, rickettsiosis) in Albania, suggesting that testing for these agents are necessary in cases with fever and hemorrhagic manifestations [6]. An outbreak of cases of CCHF occurred in Albania during 2001, repeating in the spring and summer seasons [7].

Our patient was a farmer. Both the climatic and geographic characteristics provided the appropriate environment for ticks to reproduce and these vectors spread to rural areas involved with agriculture via cattle, sheep, goats, and small mammals such as hedgehogs, hares, and rodents. Residents in these areas work in fields in the summer, a time when the ticks are in their mature form and may be actively transferred to them by these animals [8]. The incubation period for CCHF is about 2–9 days [9]. The incubation period in our case was 4 days. After a short incubation, CCHF was characterized by the systemic symptoms such as fever, hemorrhage, headache, fatigue, muscle ache, abdominal pain, nausea, and vomiting [10]. Hemorrhage develops at various parts of the body [9]. Thrombocytopenia is characteristic laboratory finding [11]. Our case also had significantly low platelet counts. Hepatomegaly has been reported to occur in one out of three patients [12]. Our patient had increased liver and spleen (hepar and lien), respectively 19 cm and 14 cm. In 1994, Fisher-Hoch *et al.* reported successful treatment of three nosocomial cases of CCHF in Pakistan with oral ribavirin [13]. Since that time, ribavirin has been used for treatment of CCHF in different parts of the world [14]. Oral ribavirin was administered within a mean of 10 days after the onset of symptoms at the dosage recommended by WHO. The intravenous form of ribavirin was not available in Albania.

## Conclusions

People in high-risk professions in endemic areas should be informed and trained on the risk of CCHF as a matter of urgency. They should go to health centers immediately following any tick bite. Vaginal bleeding is not always a gynecological problem. In Albania, these places are the mountainous areas, so people that have traveled to these areas having symptoms after a tick bite are advised to contact their doctor.

## Abbreviations

CCHF: Crimean-Congo hemorrhagic fever
CCHFV: Crimean-Congo hemorrhagic fever virus
